# Retraction Note: Overexpression of suprabasin is associated with proliferation and tumorigenicity of esophageal squamous cell carcinoma

**DOI:** 10.1038/s41598-025-30162-7

**Published:** 2025-12-08

**Authors:** Jinrong Zhu, Geyan Wu, Qingyuan Li, Hui Gong, Junwei Song, Lixue Cao, Shu Wu, Libing Song, Lili Jiang

**Affiliations:** 1 Department of Pathophysiology, Guangzhou Medical University, Guangzhou, 511436 Guangdong China; 2 Department of Experimental Research, State Key Laboratory of Oncology in Southern China, Sun Yat-Sen University Cancer Centre, Guangzhou, 510060 China; 3 Department of Biochemistry, Zhongshan School of Medicine, Sun Yat-Sen University, Guangzhou, 510080 Guangdong China; 4 Guangdong Country Garden School, Shunde, Foshan, 528312 Guangdong China; 5 Central Laboratory, Nanshan Hospital, Guangdong Medical University, Shenzhen, 518052 Guangdong China; 6 Department of Pathophysiology, Guangzhou Medical University, Guangzhou, 511436 Guangdong China

Retraction of: *Scientific Reports* 10.1038/srep21549, published online 22 February 2016

The Editors have retracted this Article.

Two images in Fig. 3 were found to overlap with two panels in Fig. 4 of another article published earlier by different authors^1^. A β-catenin blot in Fig. 5e also appears to overlap with a GAPDH blot in another article also published earlier by different authors^2^. The Editors have therefore lost confidence in the data and conclusions of this Article.
